# The Anti-Inflammatory Effects of Flavanol-Rich Lychee Fruit Extract in Rat Hepatocytes

**DOI:** 10.1371/journal.pone.0093818

**Published:** 2014-04-04

**Authors:** Ryota Yamanishi, Emi Yoshigai, Tetsuya Okuyama, Masatoshi Mori, Hiromitsu Murase, Toru Machida, Tadayoshi Okumura, Mikio Nishizawa

**Affiliations:** 1 Department of Biomedical Sciences, College of Life Sciences, Ritsumeikan University, Kusatsu, Shiga, Japan; 2 Graduate School of Science and Engineering, Ritsumeikan University, Kusatsu, Shiga, Japan; 3 Research Organization of Science and Technology, Ritsumeikan University, Kusatsu, Shiga, Japan; 4 Department of Surgery, Kansai Medical University, Hirakata, Osaka, Japan; University of Washington, United States of America

## Abstract

Flavanol (flavan-3-ol)-rich lychee fruit extract (FRLFE) is a mixture of oligomerized polyphenols primarily derived from lychee fruit and is rich in flavanol monomers, dimers, and trimers. Supplementation with this functional food has been shown to suppress inflammation and tissue damage caused by high-intensity exercise training. However, it is unclear whether FRLFE has *in vitro* anti-inflammatory effects, such as suppressing the production of the proinflammatory cytokine tumor necrosis factor α (TNF-α) and the proinflammatory mediator nitric oxide (NO), which is synthesized by inducible nitric oxide synthase (iNOS). Here, we analyzed the effects of FRLFE and its constituents on the expression of inflammatory genes in interleukin 1β (IL-1β)-treated rat hepatocytes. FRLFE decreased the mRNA and protein expression of the *iNOS* gene, leading to the suppression of IL-1β-induced NO production. FRLFE also decreased the levels of the iNOS antisense transcript, which stabilizes iNOS mRNA. By contrast, unprocessed lychee fruit extract, which is rich in flavanol polymers, and flavanol monomers had little effect on NO production. When a construct harboring the iNOS promoter fused to the firefly luciferase gene was used, FRLFE decreased the luciferase activity in the presence of IL-1β, suggesting that FRLFE suppresses the promoter activity of the *iNOS* gene at the transcriptional level. Electrophoretic mobility shift assays indicated that FRLFE reduced the nuclear transport of a key regulator, nuclear factor κB (NF-κB). Furthermore, FRLFE inhibited the phosphorylation of NF-κB inhibitor α (IκB-α). FRLFE also reduced the mRNA levels of NF-κB target genes encoding cytokines and chemokines, such as TNF-α. Therefore, FRLFE inhibited NF-κB activation and nuclear translocation to suppress the expression of these inflammatory genes. Our results suggest that flavanols may be responsible for the anti-inflammatory and hepatoprotective effects of FRLFE and may be used to treat inflammatory diseases.

## Introduction

**Figure 1:**
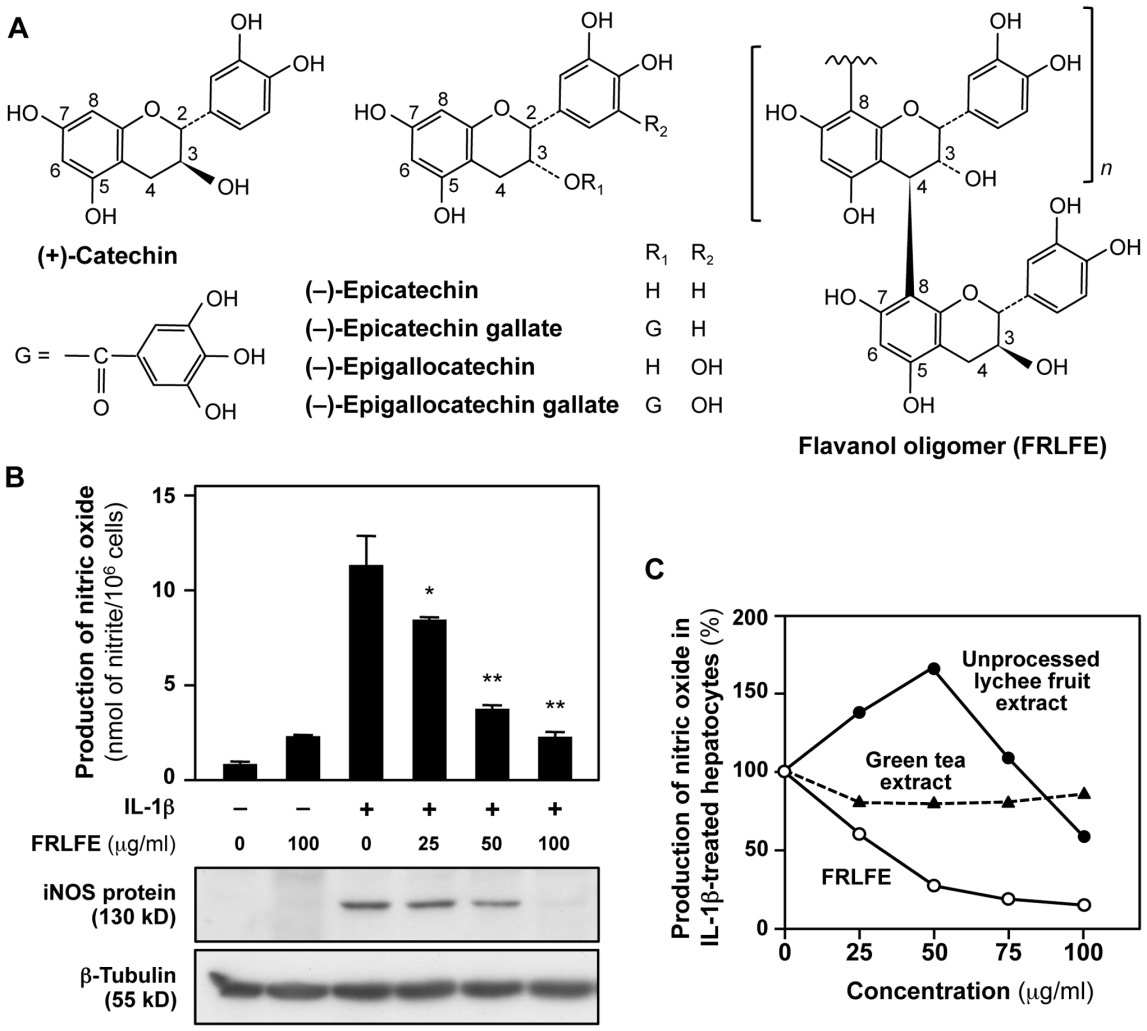
FRLFE suppresses NO production in IL-1β-treated hepatocytes. (**A**) Structures of the flavanol monomers in green tea catechins (left and center) and a flavanol polymer in FRLFE (right). Due to the two asymmetric carbons (C-2 and C-3), a flavanol monomer has four diastereoisomers, such as (+)-catechin [2*R*,3*S*] (left) and (−)-epicatechin [2*R*,3*R*] (center). (−)-Epicatechin gallate (ECG) and (−)-epigallocatechin gallate (EGCG) are galloyl esters of (−)-epicatechin and (−)-epigallocatechin (EGC), respectively. G =  galloyl group. A flavanol oligomer from FRLFE (right) was synthesized by creating a covalent bond between (+)-catechin and the lychee fruit procyanidin. (**B**) FRLFE suppresses the induction of NO production and iNOS protein expression. Rat hepatocytes were treated with or without FRLFE for 8 h. Simultaneously, 0.1 nM IL-1β was added to the cells. The NO levels in the medium were measured in triplicate (mean ± SD), and the cell extracts were immunoblotted with an anti-iNOS or anti-β-tubulin antibody (internal control). *P<0.05, **P<0.01 versus IL-1β alone. (**C**) FRLFE suppresses the induction of NO production. FRLFE, unprocessed lychee fruit extract, or green tea extract were added to the medium in the presence of 0.1 nM IL-1β. The NO levels in the medium were measured in duplicate (mean). Cytotoxicity was not observed at these concentrations (data not shown).

Fruits and vegetables are common sources of flavonoids, which are low-molecular-weight polyphenols that can be classified into six subclasses: flavonols, flavones, flavanones, flavanols (*i.e*., flavan-3-ols), isoflavones, and anthocyanidins [1]. Flavanols are a group of compounds containing flavan-3-ol as a monomer unit, and these compounds are found at high levels in a variety of fruits and beverages, for example, strawberry, lychee fruit, grape, green tea, and cacao [2], [3]. Flavanols are assumed to have health benefits, as they can augment oxidative defenses, improve vascular function, protect the central nervous system, and reduce an individual's risk of developing cancer [2], [4]. Flavanols consist of monomers (also known as catechins), dimers (dimeric procyanidins), trimers (trimeric procyanidins), oligomers (procyanidins), and polymers (tannins) [5]. Flavanol dimers, trimers, oligomers, and polymers are often collectively designated as ‘condensed tannins’. Most polyphenols contained in lychee fruit and green tea are flavanols that contain (+)-catechin or (−)-epicatechin as the monomer unit (Fig. 1A). (−)-Epicatechin and (−)-epigallocatechin (EGC) are often esterified by gallic acid to form (−)-epicatechin gallate (ECG) and (−)-epigallocatechin gallate (EGCG), respectively. EGCG is the main flavanol monomer present in green tea and has been shown to protect against cancer in rodents [6]. When [^3^H]EGCG was administered to mice using a gastric tube, this compound was widely distributed to many organs, including the digestive tract, kidney, and liver [6].

Flavanol-rich lychee fruit extract (FRLFE) is a mixture of oligomerized flavanols derived from lychee fruit (*Litchi chinensis* Sonnerat) and stabilized by the covalent binding of green tea catechins to the ends of the oligomers (Fig. 1A) [7], [8]. This dietary supplement is a phenolic mixture that contains 13–18% flavanol monomers, 14–18% dimers, and 2–6% trimers [8]–[10]. By contrast, unprocessed lychee fruit extract contains only 6.4% monomers and 9.9% dimers [9], [11]. Therefore, FRLFE is more soluble in water and has a higher bioavailability than unprocessed lychee fruit extract [12]. FRLFE is readily absorbed in the intestine and was detected in the serum of healthy volunteers [13]. Additionally, we examined the effects of FRLFE supplementation on inflammation and tissue damage in young long-distance runners during intense physical training for two months as a double-blind, randomized study [14]. Compared with the placebo group, the change in the serum interleukin 6 (IL-6) level between pre- and mid-training were significantly lower in the FRLFE group, while the change in the transforming growth factor β (TGF-β) level between pre- and post-training was significantly greater in the FRLFE group. This in vivo study suggests that FRLFE supplementation may suppress inflammation or tissue damage caused by high-intensity exercise training. However, it is still unclear how FRLFE provides such a beneficial effect at the cellular level, especially an anti-inflammatory effect on hepatocytes.

Nitric oxide (NO) plays a pivotal role as a proinflammatory mediator in various diseases [15] and is synthesized by inducible nitric oxide synthase (iNOS) in hepatocytes. The *iNOS* gene is induced by the proinflammatory cytokine interleukin 1β (IL-1β) in primary cultured rat hepatocytes, and this induction mimics an inflammatory response and liver injury in humans and animals [16], [17]. When an anti-inflammatory compound is simultaneously added with IL-1β to the culture medium, IL-1β-induced NO production and iNOS expression are suppressed (*e.g*., [18], [19]). Therefore, the suppression of IL-1β-induced iNOS expression generally correlates with anti-inflammatory activity of the compound. We demonstrated that NO is a sensitive marker of liver protection that can be used to monitor inflammatory responses to functional foods, such as the active hexose correlated compound [20], [21] and Japanese herbal medicines and their constituents [19], [22]–[24]. These compounds suppress iNOS induction at the transcriptional level through transcription factors, including nuclear factor κB (NF-κB) [25]. These constituents posttranscriptionally suppress iNOS induction using a natural antisense transcript (asRNA) that interacts with and stabilizes the iNOS mRNA [17], [26].

The asRNA is an endogenous RNA that derives from the sequence of the antisense strand of a gene, and most of the asRNAs do not encode proteins. An asRNA may posttranscriptionally regulate gene expression by interacting with the mRNA, and the asRNA itself may act in concert with various RNA-binding proteins, drugs, and herbal constituents [27]. Similarly to the iNOS asRNA, we found that the proinflammatory cytokine tumor necrosis factor α (TNF-α) is secreted from rat hepatocytes, and that TNF-α asRNA affects the stability of TNF-α mRNA [28]. Furthermore, many asRNAs are transcribed from inflammatory cytokines and chemokines, including interferon (IFN) α1, and these asRNAs affect mRNA stability by interacting with their corresponding mRNAs [29], [30]. asRNA-mediated posttranscriptional regulation is thought to be a general mechanism to modulate the stability of mRNAs involved in inflammation [27], [29]. Furthermore, several herbal constituents have been shown to suppress the expression of iNOS asRNA [19], [22]–[24]. Therefore, FRLFE is expected to be involved in the asRNA-mediated regulation of *iNOS* gene expression.

The *in vitro* effects of FRLFE on gene expression in hepatocytes have not been well studied; however, the flavanols in FRLFE are expected to affect the genes involved in inflammation, according to the results of the *in vivo* study [14]. Here, we evaluated the effects of FRLFE on the expression of the *iNOS* and inflammatory genes using rat hepatocytes. Then, the effects of unprocessed materials (*i.e*., unprocessed lychee fruit extract and green tea extract) and various flavanol monomers on the NO production were examined. We tried to elucidate the mechanisms of anti-inflammatory action of FRLFE in the *iNOS* gene expression.

## Materials and Methods

### Ethics Statement

All animal care and experimental procedures were carried out in strict accordance with the guidelines and laws of the Japanese government and were approved by the Animal Care Committee of Ritsumeikan University, Biwako-Kusatsu Campus. All surgery was performed under sodium pentobarbital anesthesia, and all efforts were made to minimize suffering.

### Materials

Lychee fruit (*Litchi chinensis* Sonnerat) collected in Guangxi Zhuang Autonomous Region, China, and green tea leaves (*Camellia sinensis* var. *viridis*) collected in Hunan Province, China, were identified by Mr. Gary Zheng (Guilin Layn Natural Ingredients Corp., Guilin, China) and processed at Guilin Layn Natural Ingredients Corp. to obtain lychee fruit extract and green tea extract, respectively. The voucher specimens were deposited at Amino Up Chemical Co., Ltd. (Sapporo, Japan) under the batch numbers LYE01-060901 (lychee fruit extract) and GTE15-080501 (green tea extract).

FRLFE (Oligonol) was produced from the lychee fruit extract and green tea extract using a patented technology process at Amino Up Chemical Co., Ltd. [10], [31]. Briefly, dried lychee fruits were extracted with 50% [volume to volume (v/v)] ethanol. The filtrate was evaporated and passed through a DIAION HP-20 column (Mitsubishi Chemical Corporation, Tokyo, Japan), and eluted with ethanol. The eluate was then evaporated to dryness yielding a dark brown powder. The resultant lychee fruit extract was mixed with green tea extract, which was then extracted with 50% (v/v) ethanol. Lychee fruit extract and green tea extract comprised about 84% and 16%, respectively, of the FRLFE preparation. The reaction mixture was heated at 60°C for 16 h, filtered through a DIAION HP-20 column, washed with water and eluted with 40% (v/v) ethanol. Evaporation of the eluate yielded a reddish brown powder, the final FRLFE product. The voucher specimen was deposited at Amino Up Chemical Co., Ltd. under the batch number OLF0810.

The characteristics of FRLFE, unprocessed lychee fruit extract, and green tea extract are presented in Table 1. FRLFE, unprocessed lychee fruit extract, and green tea extract (generous gifts by Amino Up Chemical Co., Ltd.) were dissolved in Williams' E (WE) medium (Sigma-Aldrich Corp., St. Louis, MO, USA), neutralized with sodium hydroxide, and stored at 4°C until use. (+)-Catechin, (−)-epicatechin, ECG, EGC, and EGCG were purchased from Wako Pure Chemical Industries Ltd. (Osaka, Japan) or Sigma-Aldrich Corp.

**Table 1:** Constituents of FRLFE, unprocessed lychee fruit extract, and green tea extract.

	Constituents	FRLFE*	Unprocessed lychee fruit extract**	Green tea extract*	Method***
Total polyphenols	Flavanols	≥80	≥70	≥98	Porter method + HPLC (monomers); Folin method
Flavanol monomers	Catechin, epicatechin, epicatechin gallate, and epigallocatechin gallate (EGCG)	15.0±1.1	6.2±1.4	69.9±3.6	HPLC
Flavanol dimers	Procyanidin A1, A2, B1, and B2 and epicatechin-EGCG	16.3±1.1	11.8±2.2	7.6±1.3	HPLC
Flavanol trimer	Epicatechin-procyanidin A2	4.1±0.7	5.1±1.0	0.9±0.3	HPLC
Other phenolic compounds	Other trimers, tetramers, oligomers, polymers	≥45	≥47	≥20	Calculated by the amounts#

*Ratio of weight is expressed as mean ± standard deviation in percentage (*n* = 10) ([10]; Amino Up Chemical Co., Ltd., unpublished data).

**Ratio of weight is expressed as mean ± standard deviation in percentage (*n* = 14) ([10]; Amino Up Chemical Co., Ltd., unpublished data).

***Described in the *Materials and Methods*.

# Total polyphenols – (monomers + dimers + trimer).

### Analyses of flavanols

Total polyphenols in lychee fruit extract were measured by the modified Porter method [32], which degraded proanthocyanidins to anthocyanidins in boiling water under acidic conditions. Briefly, 0.5 ml of a 50 μg/ml (w/v) solution of FRLFE was added to 1.5 ml of *n*-butanol/HCl (95∶5, v/v) and 50 μl of a 2% (w/v) solution of NH_4_Fe(SO_4_)_2_‚·12H_2_O dissolved in 2 M HCl. The reaction mixture was capped and then thoroughly mixed and heated in a water bath at 95°C for 40 min. For the analysis of individual flavanols, reversed-phase high-performance liquid chromatography (HPLC) was performed using an L-2000 series HPLC instrument equipped with a UV detector at 254 nm (Hitachi High-Technologies Corporation, Tokyo, Japan). Samples were separated by a CAPCELL PAK C_18_ column (2.0 mm internal diameter ×250 mm; Shiseido Co., Ltd., Tokyo, Japan) at 0.18 ml/min with a mobile phase of absolute methanol:1.25% (v/v) acetic acid (15∶85 to 90∶10 over 50 min). Total polyphenols in green tea extract were measured by Folin method [33] using gallic acid as a standard. Briefly, each sample (100 μl) was mixed with 500 μl of 0.2 M Folin and Ciocalteu's Phenol Reagent (Sigma-Aldrich Corp.). After the addition of 400 μl of 7.5% (w/v) Na_2_CO_3_, the mixture was incubated at 22–25°C for 1 h and then absorbance at 760 nm was measured.

### Preparation of primary cultured rat hepatocytes

Male Wistar rats were purchased from Charles River Laboratories Japan Inc. (Yokohama, Japan), housed at 21–23°C, and acclimatized prior to experimentation. Hepatocytes were isolated from the livers of the rats using collagenase perfusion [34]. Briefly, the dispersed cells were purified, resuspended in WE medium supplemented with newborn calf serum (SAFC Biosciences Inc., Lenexa, KS, USA), and seeded at a density of 1.2×10^6^ cells/dish. The cells were incubated at 37°C for 2 h, and the medium was replaced. The hepatocytes were incubated at 37°C overnight and analyzed on the next day (Day 1).

### Determination of NO levels and LDH activity

On Day 1, the hepatocytes were treated with FRLFE or another compound in the presence of rat IL-1β (PeproTech, Rocky Hill, NJ, USA) for 8 h. Nitrite (a stable metabolite of NO) levels in the culture medium were measured using the Griess method [35]. When IL-1β increased the NO in the medium, the NO level in the presence of IL-1β was set to 100%, whereas the NO level in the absence of IL-1β was set to 0%. Unless IL-1β increased the NO levels, further analyses were not performed. Gallic acid (Sigma-Aldrich Corp.), which is included in ECG and EGCG as a galloyl group, was used as a positive control to monitor the suppression of IL-1β-indcuced NO production. The half-maximal inhibitory concentrations (IC_50_) were determined (three dishes per time point) for at least three different concentrations [22]. When a compound is not cytotoxic to hepatocytes, the NO levels (%) at the concentrations are inversely proportional to log_10_[concentration] (*i.e*., dose-dependent) and thus used to determine the IC_50_ value. To monitor the cytotoxicity of the compounds, the LDH activities of the media were measured in triplicate using the LDH Cytotoxicity Detection Kits (Takara Bio Inc., Otsu, Shiga, Japan).

### Western blot analysis

Hepatocytes were treated with 0.1 nM IL-1β and 100 μg/ml FRLFE for 8 h on Day 1, and whole-cell lysates were prepared [23]. Briefly, hepatocytes (1×10^6^ cells/35-mm dish) were lysed using sample buffer (125 mM Tris-HCl, pH 6.8, 5% glycerol, 2% sodium dodecyl sulfate (SDS), and 2% 2-mercaptoethanol), subjected to SDS-polyacrylamide gel electrophoresis (PAGE), and immunoblotted onto a Sequi-Blot membrane (Bio-Rad, Hercules, CA, USA). Immunostaining was performed using primary antibodies that had been raised against rat iNOS (Thermo Fisher Scientific, Waltham, MA, USA), human NF-κB inhibitor α (IκB-α; Santa Cruz Biotechnology, Santa Cruz, CA, USA), phosphorylated IκB-α (Ser32/36 [5A5]), and rat β-tubulin (Cell Signaling Technology Inc., Danvers, MA, USA), followed by visualization with the Enhanced Chemiluminescence Blotting Detection Reagent (GE Healthcare Biosciences Corp., Piscataway, NJ, USA).

### Microarray analysis

Hepatocytes were incubated in the presence of 0.1 nM IL-1β with or without 100 μg/ml FRLFE for 2.5 h, and total RNA was purified using an RNAqueous kit (Applied Biosystems). Total RNA was labeled using an Ambion WT Expression Kit (Affymetrix Inc., Santa Clara, CA, USA) and a GeneChip WT Terminal Labeling and Controls Kit (Affymetrix Inc.) and was subjected to expression analysis using the GeneChip Rat Gene 1.0 ST Array (Affymetrix Inc.). The expression data were analyzed using the Expression Console Software (Affymetrix Inc.). Significant changes in mRNA expression were predicted by the signal ratios and Z score transformation [36]. To determine the ‘increased transcripts’ in the hepatocytes treated with FRLFE and IL-1β compared to the hepatocytes treated with IL-1β alone, we selected probe sets with a signal ratio ≥2.0 and a Z score ≥2.0. For the ‘decreased transcripts’ caused by FRLFE treatment, we selected probe sets with a signal ratio ≤0.5 and a Z score ≤−2.0.

### Reverse transcription-polymerase chain reactions (RT-PCR)

Hepatocytes were treated with 0.1 nM IL-1β and/or 100 μg/ml FRLFE. Total RNA was prepared from the hepatocytes using the Sepasol-RNA I Super G (Nacalai Tesque Inc.) and TURBO DNA-free kits (Life Technologies Corporation, Austin, TX, USA). The cDNA was reverse-transcribed in a strand-specific manner using an oligo(dT) primer for mRNA and a gene-specific sense primer for the iNOS asRNA [17], [29]. Step-down PCR was performed with paired primers [37], using glyceraldehyde-3-phosphate dehydrogenase (GAPDH) mRNA as an internal control. The mRNA levels were estimated in triplicate using real-time PCR analysis with SYBR Green I and the Thermal Cycler Dice Real Time System (Takara Bio, Otsu, Shiga, Japan) [17]. The values were normalized to the levels of GAPDH mRNA. The primers used for RT-PCR and real-time PCR are shown in Table 2.

**Table 2:** Primers used for strand-specific RT-PCR in this study.

Transcript to be detected	Sequence (5′––>3′)	RT-PCR*	Direction	cDNA (bp)**
CXCL1 mRNA	GCCAAGCCACAGGGGCGCCCGT	PCR	Forward	231
	ACTTGGGGACACCCTTTAGCATC	PCR	Reverse	
GAPDH mRNA	CCCATCACCATCTTCCAGGAGCGAG	PCR	Forward	285
	GTTGTCATGGATGACCTTGGCCAGG	PCR	Reverse	
IL-23A mRNA	CAAGGACAACAGCCAGTTCTGTT	PCR	Forward	176
	GGTGATCCTCTGGCTGGAGGAGC	PCR	Reverse	
iNOS mRNA	CCAACCTGCAGGTCTTCGATG	PCR	Forward	257
	GTCGATGCACAACTGGGTGAAC	PCR	Reverse	
iNOS asRNA#	TGCCCCTCCCCCACATTCTCT	RT	Forward	
	ACCAGGAGGCGCCATCCCGCTGC	PCR	Forward	185
	CTTGATCAAACACTCATTTTATTAAA	PCR	Reverse	
NF-κB p65 mRNA	ACCCCTTTCAAGTTCCCATAGA	PCR	Forward	262
	ACCTCAATGTCTTCTTTCTGCAC	PCR	Reverse	
NF-κB p50 mRNA	CCTGCTCCTGGAGGGTGACGCC	PCR	Forward	254
	GTATGTCAAATACCTGCCAGTTG	PCR	Reverse	
TNF-α mRNA	TCCCAACAAGGAGGAGAAGTTCC	PCR	Forward	275
	GGCAGCCTTGTCCCTTGAAGAGA	PCR	Reverse	

*The oligo(dT) primer was used for reverse transcription (RT) to synthesize the complementary DNAs (cDNAs) for each mRNA.

**The size of the cDNA fragment amplified by each pair of polymerase chain reaction (PCR) primers is shown in base pairs (bp).

# A sense primer was used for the reverse transcription of the iNOS antisense transcript (asRNA).

CXCL1, chemokine (C-X-C motif) ligand 1; GAPDH, glyceraldehyde-3-phosphate dehydrogenase; IL-23A, interleukin 23, α subunit p19; iNOS, inducible nitric oxide synthase; NF-κB, nuclear factor κB; TNF-α, tumor necrosis factor α.

### Firefly luciferase assays

Hepatocytes (3.0×10^5^ cells per dish) were transfected in duplicate with plasmid DNA using the MATra-A Reagent (IBA GmbH, Göttingen, Germany) [17]. Two plasmids were used: pRiNOS-Luc-3′UTR (1.0 μg), a luciferase reporter plasmid harboring a 1.0-kilobase iNOS promoter and the luciferase gene fused to the 3′ untranslated region (3′UTR) of the iNOS mRNA, and pCMV-LacZ (1 ng), an internal control plasmid expressing β-galactosidase driven by the cytomegalovirus enhancer/promoter [38]. The cells were cultured overnight and then treated with IL-1β and/or FRLFE for 3 h. The luciferase and β-galactosidase activities were measured using the PicaGene (Wako Pure Chemical Industries Ltd.) and Beta-Glo kits (Promega Corporation, Madison, WI, USA), respectively.

### Electrophoretic mobility shift assays (EMSA)

EMSAs were performed as previously described [23]. Briefly, nuclear extracts from the hepatocytes (4.0 μg) were mixed with 1.0 μg of poly(dI-dC). To prepare the double-stranded DNA probe, the annealed oligonucleotides harboring an NF-κB-binding site (5′- AGTTGAGGGGACTTTCCCAGGC -3′; only the sense strand is shown) were labeled with [γ-^32^P]ATP (PerkinElmer Inc., Waltham, MA, USA) and T4 polynucleotide kinase (Takara Bio Inc.). The probe was added to the nuclear extracts, which were then incubated for 20 min at 20–25°C and resolved on a 4.8% polyacrylamide gel. The gel was dried and subjected to autoradiography under an X-ray film.

### Statistical analyses

The results presented in the figures are representative of at least three independent experiments that yielded similar results. The values are presented as the mean ± standard deviation (SD). Differences were analyzed using Student's *t*-test. Statistical significance was set at *P*<0.05 and *P*<0.01.

## Results

### FRLFE efficiently suppresses NO induction in hepatocytes

To investigate the effects of FRLFE on NO induction, we added FRLFE to the culture medium of rat hepatocytes treated with IL-1β. As shown in Fig. 1B, FRLFE suppressed NO induction in the presence of IL-1β in a dose-dependent manner. Evaluation of LDH release into the medium indicated that FRLFE displayed no cytotoxicity at concentrations up to 100 μg/ml (data not shown). FRLFE effectively suppressed the IL-1β-induced NO production, with an IC_50_ value of 28.3±9.0 μg/ml (*n* = 4). Hereafter, we added FRLFE at a final concentration of 100 μg/ml, in combination with IL-1β for the subsequent experiments.

To obtain a final FRLFE product, unprocessed lychee fruit extract was mixed with green tea extract at a ratio of 84%∶16% [10]. We examined whether these unprocessed extracts (Table 1) affected the NO production, similarly to FRLFE. Unexpectedly, unprocessed lychee fruit extract increased NO production when added at a concentration of 50 μg/ml (Fig. 1C) and showed cytotoxicity at concentrations more than 100 μg/ml (data not shown). By contrast, green tea catechins extract showed only slight decreases in NO production, and thus an IC_50_ value was not determined.

Because flavanol monomers are abundant in FRLFE, we examined the effects of the monomers in FRLFE on the IL-1β-induced NO production in rat hepatocytes. As shown in Table 3, the flavanol monomers [(+)-catechin, (−)-epicatechin, ECG, EGC, and EGCG] suppressed the NO production in IL-1β-treated hepatocytes. Gallic acid was used as a positive control of the NO suppression. Among them, EGC showed the highest NO suppression activity (IC_50_  = 28.1 μg/ml), which was comparable to that of FRLFE. These results suggested that the flavanol monomers included in FRLFE may partly attribute to the NO suppression activity of FRLFE.

**Table 3:** Inhibition of nitric oxide production by the flavanol monomers.

Compound	Abbreviation	Molecular weight	IC_50_ (μM)	IC_50_ (μg/ml)
(+)-Catechin		290.27	NA	NA
(−)-Epicatechin	EC	290.27	128.5	37.3
(−)-Epicatechin gallate	ECG	442.37	75.7	33.5
(−)-Epigallocatechin	EGC	306.27	91.9	28.1
(−)-Epigallocatechin gallate	EGCG	458.37	89.4	41.0
Gallic acid (positive control)		170.12	212	36.1
Flavanol-rich lychee fruit extract	FRLFE	---	---	28.3
Unprocessed lychee fruit extract		---	---	ND
Green tea extract		---	---	NA

When IL-1β increased nitric oxide (NO) in the medium, the NO level in the presence of IL-1β was set to 100%, whereas the NO level in the absence of IL-1β was set to 0%. Gallic acid was used as a positive control to monitor the suppression of IL-1β-indcuced NO production.

IC_50_, half-maximal (50%) inhibitory concentration of NO production in IL-1β-treated hepatocytes.

NA, not applied because 50% suppression of NO production was not observed.

ND, not determined because NO levels increased.

### FRLFE suppresses the expression of the *iNOS* gene

**Figure 2:**
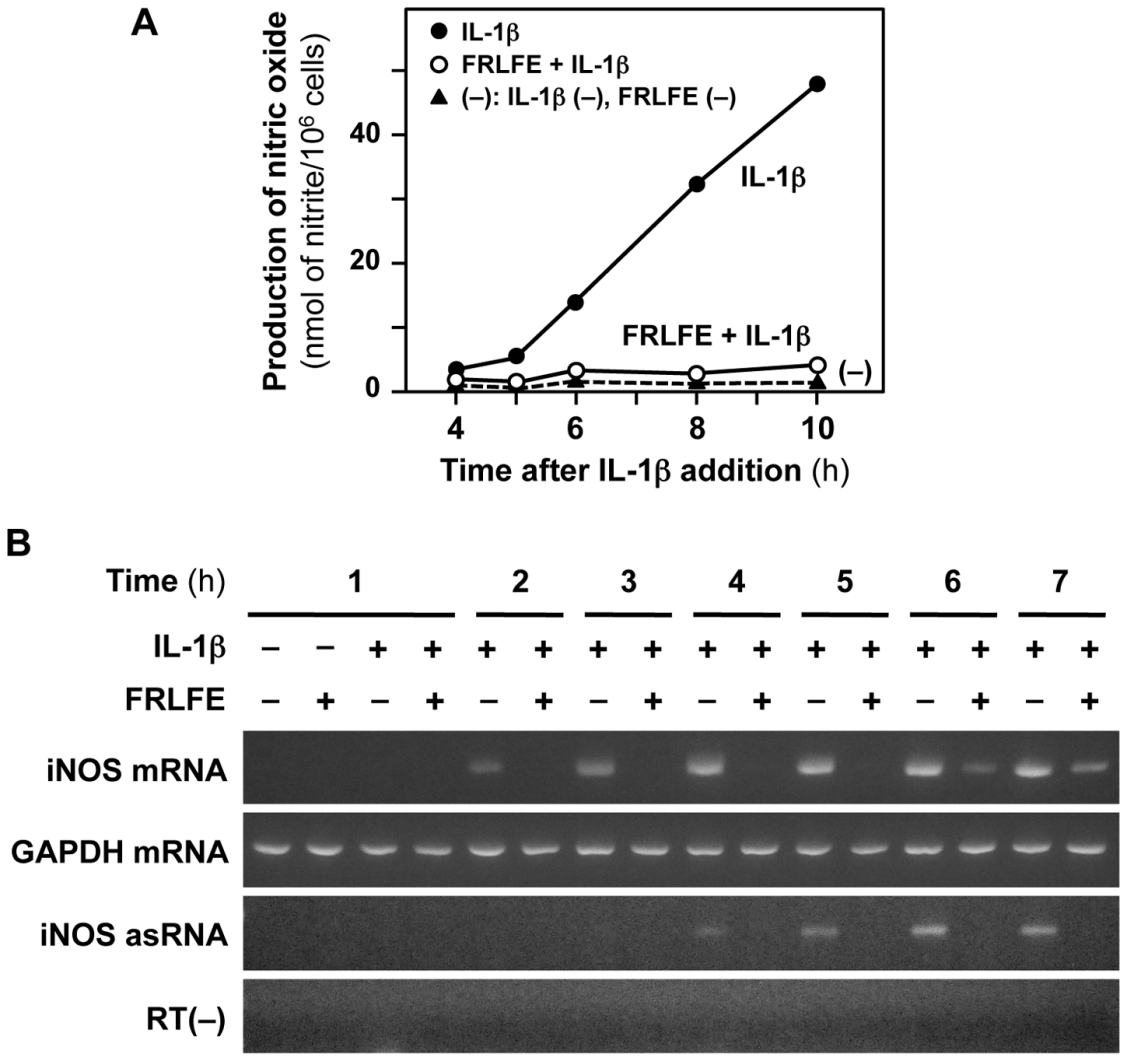
FRLFE suppresses the induction of the *iNOS* gene in the hepatocytes. (**A**) FRLFE suppresses NO induction. Hepatocytes were treated with or without IL-1β (0.1 nM) and/or FRLFE (100 μg/ml) for the indicated times. The NO levels in the medium were measured in duplicate. (**B**) FRLFE decreases the expression levels of both iNOS mRNA and its asRNA. Hepatocytes were treated with IL-1β and/or FRLFE, and total RNA from the cells was analyzed using strand-specific RT-PCR. The iNOS mRNA and its asRNA, as well as GAPDH mRNA (internal control), were detected by agarose gel electrophoresis of the PCR products. RT(−) indicates the negative PCR control without RT, which was used to monitor contamination with genomic DNA.

Next, we analyzed the effects of FRLFE on the expression of the *iNOS* gene in hepatocytes. The induction of NO production and iNOS expression in IL-1β-treated hepatocytes mimics an inflammatory response and liver injury [16], [17]. Because FRLFE suppressed the NO production in IL-1β-treated hepatocytes, FRLFE is expected to suppress the *iNOS* gene expression. As shown in Fig. 2A, FRLFE markedly decreased the IL-1β-induced NO production in a time-dependent manner. Western blot analyses indicated that FRLFE dose-dependently decreased iNOS protein expression in the hepatocytes (Fig. 1B). RT-PCR analyses revealed that FRLFE markedly reduced the IL-1β-induced iNOS mRNA expression (Fig. 2B). These results imply that FRLFE suppressed the induction of *iNOS* gene expression at the transcriptional level.

Furthermore, strand-specific RT-PCR showed that FRLFE prominently decreased the levels of iNOS asRNA (Fig. 2B). The iNOS asRNA interacts with and stabilizes the iNOS mRNA [17], [26]. Because the reduction of the iNOS asRNA levels leads to decreased iNOS mRNA stability, these results imply that FRLFE may also regulate the mRNA levels of iNOS at a posttranscriptional level.

### FRLFE decreases iNOS promoter activity

**Figure 3:**
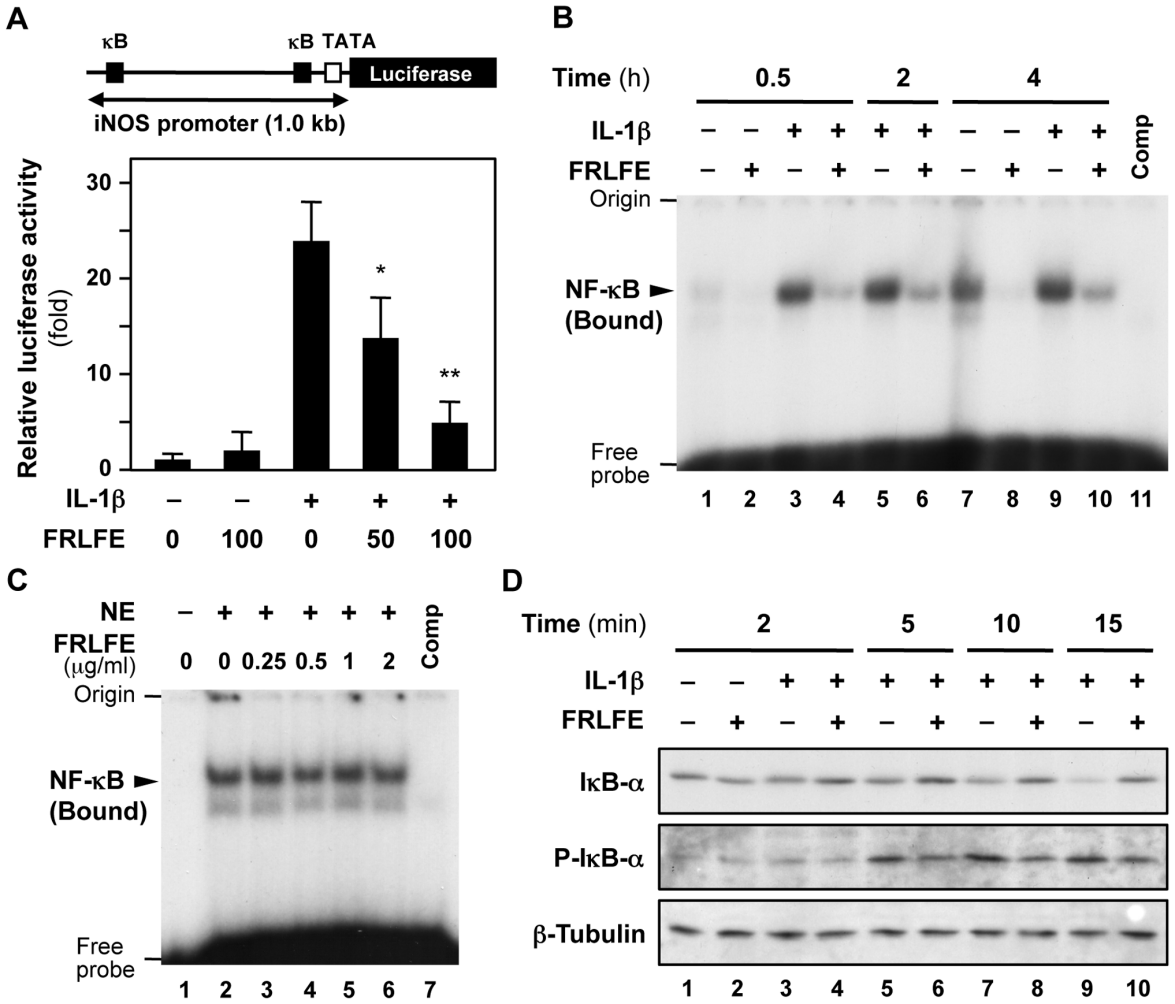
The effects of FRLFE on the NF-κB-dependent transcription of the *iNOS* gene. (**A**) FRLFE decreases the iNOS promoter activity. Hepatocytes were transfected with an iNOS promoter–luciferase construct (pRiNOS-Luc-3′UTR; top) and pCMV-LacZ (internal control) and were subsequently treated with IL-1β and/or FRLFE. κB, NF-κB-binding site; TATA, TATA box. The luciferase activity in the transfected cells was normalized to the β-galactosidase activity, and the fold activation was calculated by dividing the normalized luciferase activity by the luciferase activity in the presence of IL-1β alone. The data represent the mean ± SD (*n* = 3). *P<0.05, **P<0.01 versus IL-1β alone. (**B**) Nuclear extracts from FRLFE-treated hepatocytes decrease the DNA-binding activity to an NF-κB-binding site. Nuclear extracts were prepared from the cells and were analyzed using an EMSA to detect the NF-κB that was bound to a radiolabeled DNA probe harboring an NF-κB-binding site (κB). Competitor (Comp), cold DNA probe that was added to the reaction mixture at 100-fold molar excess to the radiolabeled probe. (**C**) FRLFE does not directly inhibit the DNA-binding activity of nuclear NF-κB. To induce NF-κB, hepatocytes were treated with IL-1β alone for 0.5 h, and a nuclear extract (NE) was prepared from these cells. The nuclear extract was directly mixed with FRLFE and analyzed using an EMSA to detect the NF-κB that was bound to the DNA probe harboring an NF-κB-binding site, similarly to (B). (**D**) FRLFE decreases the phosphorylation of IκB-α. Hepatocytes were treated with IL-1β and/or FRLFE for the indicated times. Hepatocyte extracts were immunoblotted with an anti-IκB-α, anti-phosphorylated IκB-α (p-IκB-α), or anti-β-tubulin antibody (internal control).

To further analyze the mechanism of transcriptional regulation, we performed reporter assays using an iNOS promoter–firefly luciferase construct (Fig. 3A). Because luciferase transcription is driven by the *iNOS* gene promoter, the luciferase activity represents the promoter activity and thus corresponds to iNOS mRNA synthesis [38]. As shown in Fig. 3A, IL-1β increased the luciferase activity, whereas FRLFE significantly reduced the luciferase activity in the presence of IL-1β, demonstrating that FRLFE reduced the promoter activity of the *iNOS* gene.

### FRLFE decreases the nuclear translocation of NF-κB

To clarify the FRLFE-mediated transcriptional suppression, we investigated NF-κB, which plays a pivotal role in inflammation and iNOS induction [25], [39]. IL-1β stimulates the degradation of IκB proteins after phosphorylation by the IκB kinase (IKK); this leads to the activation of NF-κB, resulting in its translocation from the cytoplasm into the nucleus and its association with gene promoters. Therefore, nuclear extracts of FRLFE-treated hepatocytes were analyzed using an EMSA with a radiolabeled DNA probe harboring an NF-κB-binding site (Fig. 3B). The results showed decreased band signals in the presence of FRLFE and IL-1β from 0.5–4 h after the addition of IL-1β (lanes 4,6,10), suggesting that FRLFE in the culture medium decreased the DNA-binding activity of NF-κB.

We further examined whether FRLFE directly inhibited the binding of NF-κB to the DNA probe. Because IL-1β induced NF-κB expression, nuclear extracts were prepared from IL-1β-treated hepatocytes. FRLFE was added to the reaction mixture that included the IL-1β-treated nuclear extract and analyzed using an EMSA (Fig. 3C). The results revealed that band signals of DNA-bound NF-κB did not change in the presence of FRLFE, suggesting that FRLFE did not directly inhibit the binding of NF-κB to its binding site. Together, FRLFE may inhibit the translocation of NF-κB into the nucleus.

### FRLFE reduces the phosphorylation of IκB-α

Next, we examined whether FRLFE affected the phosphorylation and degradation of IκB-α, which regulates the translocation of NF-κB into the nucleus. As shown in Fig. 3D, FRLFE markedly decreased the phosphorylation of IκB-α following stimulation with IL-1β for 5–15 min (lanes 6,8,10), whereas FRLFE slightly increased the IκB-α levels. These data indicate that FRLFE may inhibit the transcription of the *iNOS* gene by decreasing IκB-α phosphorylation, thus decreasing the levels of nuclear NF-κB.

**Figure 4:**
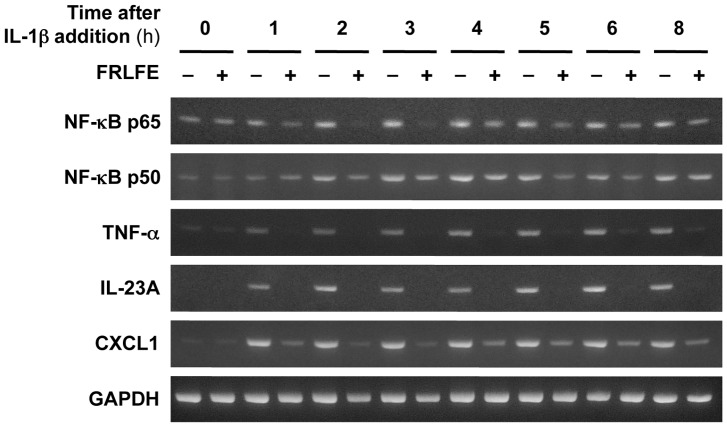
FRLFE suppresses the mRNA induction of inflammatory genes. Hepatocytes were treated with IL-1β and/or FRLFE for the indicated times, and total RNA was subjected to RT-PCR, followed by agarose gel electrophoresis to resolve the PCR products. The mRNAs encoding NF-κB p65 and p50 subunits, TNF-α, CXCL1, IL-23A, and GAPDH (internal control) were analyzed.

We further measured the mRNA levels of the NF-κB subunits (p65 and p50) that activate the *iNOS* gene [25], [39]. As shown in Fig. 4 and Table 4, RT-PCR indicated that FRLFE markedly reduced IL-1β induction of both p65 and p50 mRNAs. These results suggest that FRLFE reduced not only the nuclear NF-κB levels but also the mRNA levels of its subunits.

**Table 4:** Transcripts reduced by FRLFE in rat hepatocytes.

Gene symbol	Gene description	Signal ratio (FRLFE+IL-1β/IL-1β)*	mRNA decrease by FRLFE**	asRNA	Reference
Nos2	iNOS	0.060	0.000	(+)	17
Il23a	IL-23A	0.103	0.001	(+)	29
Cx3cl1	CX3CL1	0.109	Not determined	(+)	29
Ccl20	CCL20	0.123	Not determined	(+)	29
Ccl2	CCL2	0.128	Not determined	(+)	29
Tlr2	toll-like receptor 2	0.275	Not determined	(+)	29
Tnf	TNF-α	0.281	0.005	(+)	28
Cd69	Cd69 molecule	0.284	Not determined	(+)	29
Cxcl1	CXCL1	0.321	0.005	(+)	29
Psmb10	PSMB10	0.449	Not determined	(+)	29
Ltb	lymphotoxin β	0.484	Not determined	(+)	29
Sema4a	semaphorin 4a	0.499	Not determined	(+)	29
Nfkb1	NF-κB, p50 subunit	0.660	0.041	(+)	29
Nfkbia	IκB-α	0.690	0.033	(+)	29
Rela	NF-κB, p65 subunit	0.877	0.219	(+)	29

*The fold-change in signal ratios observed by microarray analysis of the mRNA levels at 2.5 h is indicated.

**mRNA decrease represents the ratio of the mRNA levels determined by real-time RT-PCR (FRLFE + IL-1β versus IL-1β) at 4 h. The mRNA level of IL-1β was set to 100%.

(+), asRNA was experimentally detected.

iNOS, inducible nitric oxide synthase; IL-23A, interleukin 23, α subunit p19; CX3CL1, chemokine (C-X_3_-C motif) ligand 1; CCL, chemokine (C-C motif) ligand; TNF-α, tumor necrosis factor α; CXCL1, chemokine (C-X-C motif) ligand 1; PSMB10, proteasome subunit, β type 10; NF-κB, nuclear factor κB; IκB-α, nuclear factor κB inhibitor α.

### FRLFE suppresses IL-1β-induced expression of many inflammatory genes

To determine the mRNA expression changes caused by FRLFE addition, microarray analyses were performed using 29,214 probe sets. Our previous microarray analyses demonstrated that there were 592 inducible transcripts that significantly increase in IL-1βtreated rat hepatocytes [29]. These increased transcripts include mRNAs encoding cytokines and chemokines that are involved in inflammation. It is possible that FRLFE suppresses the transcripts that are induced by IL-1β. Therefore, total RNA was prepared from rat hepatocytes treated with both FRLFE and IL-1β or with IL-1β alone and subjected to a microarray analysis. Then, we compared the mRNA expression profiles of the hepatocytes treated with both FRLFE and IL-1β to those treated with IL-1β alone. The significant changes in mRNA expression were predicted using the signal ratios and Z scores [36], as described in the *Materials and Methods*. Signals showing a significant decrease (signal ratio ≤0.5) were detected for 279 transcripts, which were assumed to be transcripts that were suppressed by FRLFE treatment (data not shown). By contrast, only 58 transcripts increased following FRLFE (signal ratio ≥2.0) treatment.

Among the 279 transcripts, there were several transcripts that were prominently decreased by FRLFE, such as iNOS, TNF-α, the α subunit p19 of IL-23 (IL-23A), and chemokine (C-X-C motif) ligand 1 (CXCL1) (Table 4). To confirm the FRLFE-induced changes in expression, we performed RT-PCR analysis for these genes involved in inflammation. As shown in Fig. 4, the results showed that FRLFE significantly suppressed the induction of these mRNAs. Real-time RT-PCR confirmed that FRLFE markedly suppressed the IL-1β-induced induction of these mRNAs (Table 4). Interestingly, these suppressed genes, including the *iNOS* and *Tnf* genes, harbor NF-κB-binding site(s) in their promoters (data not shown). Furthermore, all of the genes in Table 4 transcribe asRNAs that correspond to the 3′ untranslated regions of their mRNAs [29].

## Discussion

The present study clearly demonstrates that FRLFE suppressed the levels of the proinflammatory mediator NO and the expression of iNOS in IL-1β-treated rat hepatocytes (Figs. 1B and 2A). Furthermore, FRLFE decreased the levels of mRNA encoding the proinflammatory cytokine TNF-α (Fig. 4). The *in vitro* system using rat hepatocytes mimics an inflammatory response and liver injury to produce NO and TNF-α in response to IL-1β [15], [16], [28]. Therefore, the suppression of both iNOS and TNF-α induction suggests that FRLFE has anti-inflammatory and hepatoprotective effects. Because FRLFE markedly suppressed IL-1β-induced iNOS expression, it is possible that the flavanols in FRLFE are responsible for the pharmacological actions of FRLFE, such as NO suppression.

The flavanol-rich polyphenols in FRLFE suppressed IL-1β-induced NO production in rat hepatocytes. Flavanols at various degrees of polymerization are included in FRLFE, including monomers, dimers, and trimers (Table 1). Unprocessed lychee fruit extract, which was a mixture that included less than 20% flavanol monomers and dimers, instead enhanced NO production (Fig. 1C). In contrast, green tea extract, which included about 80% monomers and dimers, showed only slight decreases of NO production. Among the monomers in green tea extract, EGC showed the highest NO suppression activity, with an IC_50_ value of 28.1 μg/ml (Table 3). Given that the IC_50_ value of FRLFE was 28.3 μg/ml and the content of flavanol monomers in FRLFE was 15.0%, the concentration of flavanol monomers was assumed to be 4.2 μg/ml. If all the flavanol monomers in FRLFE are EGC, the NO suppression activity of FRLFE cannot be entirely attributed to the activity of EGC. On the other hand, FRLFE included more flavanol monomers and dimers than unprocessed lychee fruit extract (Table 1). Therefore, it seems plausible that flavanols longer than monomers are also the candidates responsible for the NO suppression activity of FRLFE.

The polymerization level of the flavanols may correlate with their physiological functions. In human peripheral blood mononuclear cells, small (flavanol monomer to tetramer) fractions of cocoa decrease IL-1β mRNA levels, whereas large (pentamer to decamer) fractions increase these levels [40]. Yamashita *et al*. administered four flavanols from cacao liquor (cocoa mass) to mice: (−)-epicatechin, procyanidin B2 (dimer), procyanidin C1 (trimer), and cinnamtannin A2 (tetramer) [41]. Only cinnamtannin A2 prominently increased the levels of glucagon-like peptide-1 (GLP-1) and insulin secretion in the mouse plasma. These data suggest that flavanols at different polymerization levels can cause differential responses in cytokine and incretin production. These findings also support the possibility that flavanol oligomers at optimum polymerization levels (*i.e*., intermediate chain oligomers) are most effective at suppressing NO induction.

The absorption and dynamics of flavanols are reported. Apple flavanols (monomers to pentamers) were detected in rat plasma after oral administration [32]. Real-time imaging of biodistribution of EGCG was performed by positron emission tomography [42]. When [^11^C]methyl-EGCG was orally or intravenously administered to rats, images of [^11^C]methyl-EGCG was quantitatively detected in the liver. Furthermore, flavanols were metabolized to the glucuronide in the liver and/or to methyl conjugates in the intestinal mucosa [32]. When hazelnut skin extract (rich in flavanols) was orally administered, the metabolites of flavanol monomers were detected in the liver, and the concentration of methyl catechin glucuronide was about 10 nmol/g tissue [43]. Accordingly, the concentration of flavanol monomers in the liver is roughly estimated to be higher than 10 μM. This expected monomer concentration in the liver would be comparable to that in the present *in vitro* study using rat hepatocytes.

**Figure 5:**
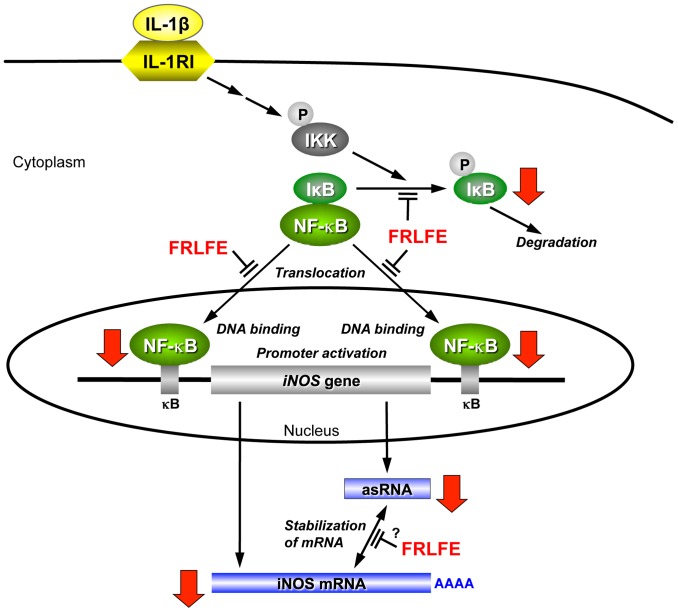
FRLFE suppresses iNOS induction by the IL-1β signaling pathway. A pathway to activate the *iNOS* gene and the action of FRLFE are schematically depicted. The bold arrows indicate the decreases caused by FRLFE in this study. The proinflammatory cytokine IL-1β binds to its receptor (type I IL-1 receptor, IL1R1) to activate NF-κB through the IκB kinase (IKK) signaling pathway [23], [25]. Activated IKK phosphorylates IκB-α, resulting in the degradation of IκB-α. A circled P denotes protein phosphorylation. Active NF-κB enters into the nucleus, binds to the *iNOS* gene promoter (κB sites), and activates transcription. FRLFE inhibits the phosphorylation of IκB-α and the nuclear translocation of NF-κB, resulting in a decrease in the nuclear levels of NF-κB. Because NF-κB also regulates the transcription of the iNOS asRNA, FRLFE reduces the level of iNOS asRNA, which interacts with and stabilizes the iNOS mRNA [17]. Furthermore, FRLFE may interfere with the iNOS mRNA–asRNA interaction at a posttranscriptional level. Therefore, FRLFE significantly decreases the level of iNOS mRNA.

How does FRLFE suppress so many genes involved in inflammation? NF-κB primarily regulates the expression of both iNOS mRNA and its asRNA at the transcriptional level [17]. FRLFE decreased the promoter activity of the *iNOS* gene (Fig. 3A), and FRLFE in the medium significantly reduced the DNA-binding activity of NF-κB in the nucleus (Fig. 3B). However, FRLFE did not directly inhibit the binding of NF-κB to its binding sites (Fig. 3C). Accordingly, these results suggested that FRLFE may inhibit the translocation of NF-κB into nucleus. Furthermore, the phosphorylation of IκB-α may be a target of FRLFE (Fig. 3D), which led to a decrease in the nuclear NF-κB levels (Fig. 5). There are three reports that support the involvement of flavanols in the inhibition of NF-κB activation. First, (+)-catechin, (−)-epicatechin and flavanol dimers from cocoa inhibit NF-κB activation in T lymphocytes [44]. These flavanols inhibit the binding of NF-κB to the DNA, as well as inhibit IKK activation, leading to the suppression of IL-2 production. Second, theaflavins, which are produced in black tea through the oxidative polymerization of two flavanol molecules, suppress iNOS induction by preventing NF-κB activation in macrophages [45]. Third, nobiletin, which is a polymethoxylated flavone found in citrus fruits, also suppresses iNOS expression and decreases the nuclear NF-κB levels in hepatocytes [24]. All of these data support the idea that flavanols in FRLFE may inhibit NF-κB activation by affecting the IκB-α phosphorylation and the NF-κB translocation into the nucleus. NF-κB-binding sites are present in the promoter of iNOS mRNA and the promoter for iNOS asRNA (*i.e*., 3′ flanking sequence of the *iNOS* gene) [17]. Therefore, FRLFE decreases the NF-κB level in the nucleus, leading to the reduction of both iNOS mRNA and its asRNA.

Using a similar mechanism, FRLFE may suppress the NF-κB-driven inflammatory genes shown in Table 4. NF-κB is involved in inflammation [25], and NF-κB-binding sites are frequently present in the promoter of the genes encoding the inflammatory cytokines, such as TNF-α [28]. However, it is unclear whether the promoters of their asRNAs harbor the NF-κB-binding sites, because most of the antisense promoters except the iNOS asRNA promoter have been not well investigated. Therefore, FRLFE may at least inhibit NF-κB to suppress transcription of the inflammatory cytokine mRNAs. The anti-inflammatory and hepatoprotective effects of the flavanols in FRLFE via NF-κB may be used to treat inflammatory diseases.

asRNAs are often transcribed from many inducible genes encoding iNOS, inflammatory cytokines and chemokines, such as TNF-α and IFN-α1 [17], [28]–[30]. The mRNA–asRNA interactions are assumed to be an essential mechanism of posttranscriptional gene regulation [27], [46], [47]. Indeed, the iNOS asRNA stabilizes the iNOS mRNA by interacting with the mRNA [17], [26]. The asRNA level is lower than its mRNA level; for example, the mRNA/asRNA ratios are 7, 30, and 100 for *iNOS*, *IFN-α1*, and *P53* genes, respectively [17], [30], [48]. To explain how a low-copy-number asRNA affect stability of its mRNA [49], we have proposed a ‘*recycling*’ model [27]. The asRNA is assumed to trigger a conformational change and partial destabilization of mRNA. These changes may affect the accessibility of RNA-binding proteins, resulting in the recruitment of a stabilizing protein. The stabilizing protein(s) can then promote protein-protein interactions to form an mRNA-asRNA-protein complex, which stabilizes the mRNA by prohibiting access of the enzymes that degrade mRNA. Finally, the asRNA is released from the complex and then recycled to stabilize another mRNA molecule. Accumulating reports have confirmed that the asRNA is involved in the regulation of mRNA stability [17], [26], [28]–[30].

Accordingly, the iNOS mRNA–asRNA interaction may be affected by the flavanols in FRLFE, thereby suppressing iNOS expression. Because the FRLFE-mediated decreases in iNOS mRNA levels were correlated with the iNOS asRNA levels (Fig. 2B), FRLFE may suppress iNOS expression by inhibiting both NF-κB activation and the iNOS mRNA–asRNA interaction (Fig. 5). Recently, it was reported that EGCG directly binds to RNA [50] and that apigenin (hydroxylated flavone) can bind RNA with high affinity [51]. These results support the possibility that the flavanols in FRLFE interfere with mRNA–asRNA interactions by binding to the mRNA and/or asRNA, leading to the suppression of the inflammatory cytokines and chemokines.

A CCCH-type zinc-finger protein regnase-1 (also known as Zc3h12a) is a ribonuclease that destabilizes mRNAs encoding IL-6, IL-12 p40 subunit, and regnase-1 itself [52]. Regnase-1 recognizes the conserved stem-loop structure for the regnase-1 responsive elements of these 3′UTR and degrades these mRNAs. If the regnase-1 responsive element is present in the 3′UTR region that interacts with the asRNA, the asRNA may block the action of regnase-1. Because a regnase-1 responsive element was not found in the iNOS mRNA, it is ruled out that regnase-1 is not involved in the iNOS mRNA stability. It is unknown whether regnase-1 may regulate the stability of mRNAs encoding other inflammatory cytokines.

In conclusion, FRLFE suppressed the expression of inflammatory genes, resulting in anti-inflammatory effects through its inhibition of NF-κB activation and mRNA–asRNA interactions. The flavanol oligomers in FRLFE may be responsible for the observed anti-inflammatory effects. These data support the possibility that the flavanols in FRLFE can be used to treat inflammatory diseases.
